# Cell Wall Loosening in the Fungus, *Phycomyces blakesleeanus*

**DOI:** 10.3390/plants4010063

**Published:** 2015-01-21

**Authors:** Joseph K. E. Ortega, Jason T. Truong, Cindy M. Munoz, David G. Ramirez

**Affiliations:** Bioengineering Laboratory, Mechanical Engineering Department, University of Colorado Denver, Denver, CO 80217, USA; E-Mails: jcttruong@gmail.com (J.T.T.); Cindy.M.Munoz@ucdenver.edu (C.M.M.); ramiredg@gmail.com (D.G.R.)

**Keywords:** *Phycomyces*, expansive growth, wall loosening, chemorheology

## Abstract

A considerable amount of research has been conducted to determine how cell walls are loosened to produce irreversible wall deformation and expansive growth in plant and algal cells. The same cannot be said about fungal cells. Almost nothing is known about how fungal cells loosen their walls to produce irreversible wall deformation and expansive growth. In this study, anoxia is used to chemically isolate the wall from the protoplasm of the sporangiophores of *Phycomyces blakesleeanus*. The experimental results provide direct evidence of the existence of chemistry within the fungal wall that is responsible for wall loosening, irreversible wall deformation and elongation growth. In addition, constant-tension extension experiments are conducted on frozen-thawed sporangiophore walls to obtain insight into the wall chemistry and wall loosening mechanism. It is found that a decrease in pH to 4.6 produces creep extension in the frozen-thawed sporangiophore wall that is similar, but not identical, to that found in frozen-thawed higher plant cell walls. Experimental results from frozen-thawed and boiled sporangiophore walls suggest that protein activity may be involved in the creep extension.

## 1. Introduction

Cellular expansive growth is central to the development, morphogenesis and sensory responses of plants, algae and fungi. Plant, algal and fungal cells have walls. These cells regulate the mechanical behavior of their walls during expansive growth to control the growth rate, morphogenesis and growth responses to environmental stimuli [1,2,3]. During expansive growth, water uptake produces turgor pressure that deforms the cell wall. Walls exhibit both irreversible and reversible (elastic) deformation during expansive growth [4,5,6]. The wall deformation behavior is similar to that of a Maxwell–Bingham viscoelastic model, which consists of a dashpot (filled with a Bingham fluid) in series with an elastic spring [4,6]. The constitutive equation (stress-strain relationship) for a Maxwell–Bingham viscoelastic model, Equation (1), describes the strain rate as equal to the sum of the irreversible extension rate of the dashpot filled with a Bingham fluid and the reversible (elastic) extension rate of a spring [4].

(1) $$\frac{1}{L}\frac{dL}{dt}=\frac{1}{µ}\;\left(\sigma \;-\sigma_{C}\right)+\;\frac{1}{E}\frac{d\sigma }{dt}$$

*Strain rate = irreversible extension rate of a dashpot + reversible extension rate of a spring*

where *L* is the length of the viscoelastic model, *t* is the time, *µ* is the dynamic viscosity of the Bingham fluid, *σ* is the stress, *σ*_C_ is the critical stress of the Bingham fluid and *E* is the longitudinal elastic modulus of the spring. The term, (d*L*/d*t*)/*L*, is the strain rate, and the term, (*σ* − *σ*_C_)/*µ*, is the irreversible extension rate of the dashpot filled with a Bingham fluid, which behaves like a Newtonian fluid after a critical stress, *σ*_C_, is exceeded. The term, (d*σ*/d*t*)/*E*, is the reversible (elastic) extension rate of the spring.

Both irreversible and reversible wall deformations are required for expansive growth. Reversible wall deformation is responsible for most of the turgor pressure production during water uptake. Irreversible wall deformation is responsible for the permanent increase in cell volume. This wall deformation behavior is implicit in the augmented growth equation, Equation (2), that was derived from the constitutive equation for a Maxwell–Bingham model, Equation (1), and which provides a quantitative mathematical description of the expansive growth rate as a function of the wall deformation rate of cells with walls [4].

(2) $$\frac{1}{V}\frac{dV}{dt}=\phi \;\left(P-P_{C}\right)+\;\frac{1}{\varepsilon }\frac{dP}{dt}$$

*Volume expansion rate = irreversible wall deformation rate + elastic wall deformation rate*

where *V* is the volume of the cell wall chamber, *t* is the time, *ϕ* is the relative wall extensibility, *P* is the turgor pressure, *P*_C_ is the critical turgor pressure and *ε* is the volumetric elastic modulus. The term, d*V*/*V*d*t*, represents the relative rate of change in volume of the cell wall chamber; the term, *ϕ* (*P* − *P*_C_), represents the relative irreversible wall deformation rate; and the term, (d*P*/d*t*)/*ε*, represents the relative reversible (elastic) wall deformation rate.

The augmented growth equation has been adapted and experimentally validated for diffuse growth, tip growth, intercalary growth, cellular growth within tissue and growth responses to environmental stimuli [2,5,6,7]. It is hypothesized that chemistry within the wall breaks and makes load-bearing bonds between wall polymers to loosen and harden the wall and regulate temporal and spatial irreversible and elastic wall deformations (chemorheology) during expansive growth and morphogenesis [1,2,5,6,7]. Furthermore, it is hypothesized that wall chemistry and chemorheological processes control the magnitude and behavior of the inclusive biophysical variables within the augmented growth equation to regulate the expansive growth rate [1,2,5,6,7]. Interestingly, the augmented growth equation was recently derived from fundamental thermodynamic principles as a particular case of a more general growth equation [8], and the evolution of *ϕ* and *ε* as a function of d*V*/*V*d*t* was correctly predicted.

Investigations with higher plant tissue provide experimental evidence indicating acidic pH initiates wall chemistry that promotes wall loosening [9]. It is shown that low pH produces continuous deformation (creep) of frozen-thawed cell walls of higher plant cells when a constant stress is applied [10,11]. Thus, a chemorheological process promotes creep in frozen-thawed cell walls. Investigators have identified and isolated pH-dependent proteins (expansins) that mediate the wall loosening and creep in frozen-thawed plant tissue [1,11]. Expansins and their genes have been found in higher plants, fungi and bacteria [1,12]. Importantly, expansins have been shown to promote creep and irreversible wall deformation in frozen-thawed tissue from many different plants and are hypothesized to play a major role in the expansive growth of higher plants [1]. Endogenous wall enzymes are also hypothesized to play a role in the expansive growth of higher plants (e.g., xyloglucan endotransglycosylase/hydrolase and endo-β-1,4-glucanases), but they have not been shown to promote creep in frozen-thawed cell walls [1,13]. Experimental evidence indicates that another chemorheological process (calcium pectate cycle) plays a major role in the expansive growth of pollen tubes [14] and algae [15] and may play a role in the expansive growth of higher plant cells [3,8].

Importantly, almost nothing is known about the mechanism of wall loosening in fungal cell walls, although a considerable amount is known about fungal cell wall structure, synthesis and assembly [16,17,18]. In the current study, experiments are conducted to obtain insight into the wall loosening mechanism of the large single-celled sporangiophores of the fungus, *Phycomyces blakesleeanus* [17,18]. The cell wall mechanical properties of these sporangiophores have been studied extensively during expansive growth and exhibit wall deformation characteristics that are similar to those of higher plant cell walls [2,6,7]. It was shown that their walls exhibit deformation behavior similar to that of a Maxwell-Bingham viscoelastic model and that the augmented growth equations accurately describe their expansive growth rate [2,4,6,7].

In the present experimental investigation, anoxia is used to chemically isolate the wall from the protoplasm of the sporangiophores of *P. blakesleeanus*. Anoxia terminates the sporangiophore’s metabolism [17,19] and essentially stops the export of wall polymers and materials from the protoplasm to the wall, thus chemically isolating the wall. The experimental results provide direct evidence of the existence of chemistry and chemorheology within the fungal wall that are responsible for wall loosening, irreversible wall deformation and elongation growth.

In addition, constant-tension extension experiments are conducted on frozen-thawed sporangiophore walls to obtain insight into the wall chemistry and chemorheology that produce irreversible wall deformation. It is found that a decrease in pH to 4.6 produces creep in frozen-thawed sporangiophore walls, similar to higher plant cells. Frozen-thawed sporangiophore walls that were boiled for 15 s before constant-tension extension experiments did not exhibit creep, again similar to higher plant cells. This inactivation of endogenous wall-loosening activity may suggest mediation by an expansin-like protein and/or mechanism. However, unlike higher plant cells, the creep of the sporangiophores’ walls continues for minutes, not hours. This result may indicate that the chemistry and chemorheology that produce creep and wall loosening in the sporangiophore’s wall may be fundamentally different from that in higher plant cells.

## 2. Results

### 2.1. Anoxia Experiments

**Figure 1:**
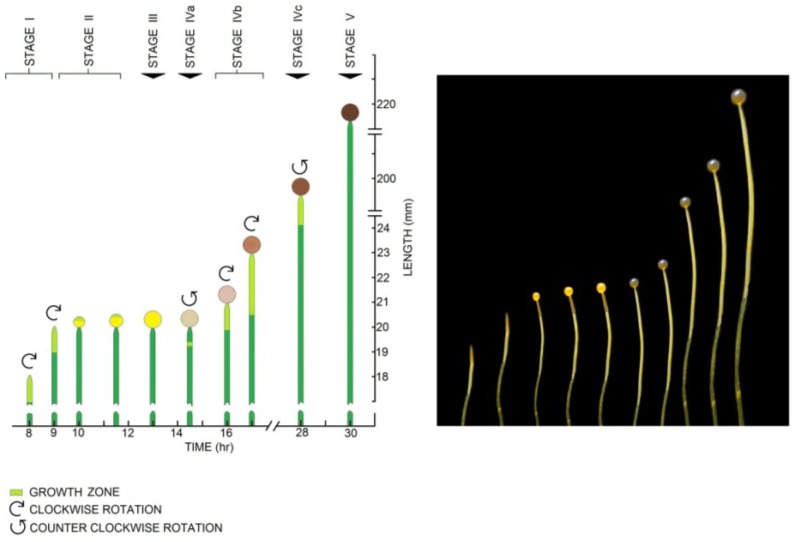
The schematic illustration (**left**) and corresponding photograph (**right**) show the developmental stages of the sporangiophore of *P. blakesleeanus*. Sporangiophore development is divided into five stages (Stages I, II, III, IV and V), and Stage IV is further divided into three sub-stages (IVa, IVb and IVc). The sporangiophores exhibit expansive growth and irreversible wall deformation in a region termed the “growth zone”. The growth zone is located at the apical tip (tip growth) in Stage I and Stage II and adjacent to the sporangium (intercalary growth) in Stage IV. The Stage I sporangiophore is a single pointed cell that grows longitudinally at the apical tip in the growth zone, 1–2 mm in length. In the schematic, the growth zone is shown as light green and the non-growing stalk is dark green. Clockwise rotation (when viewed from above) and elongation growth occur concurrently during Stage I, producing left-handed helical growth. Stage II begins with spherical growth at the apical tip without elongation and rotation growth. The diameter of the sporangium continues to increase until Stage III, where the diameter is constant (~0.5 mm). During Stage III, there is no visible expansive growth. Stage IVa begins with elongation growth concurrent with counter-clockwise rotation growth (right-handed helical growth) in a short growth zone located approximately 0.6 mm below the sporangium. The growth zone, elongation growth rate and rotation growth rate increase in magnitude as the right-handed helical growth continues for approximately one hour. Then, the rotation rate gradually decreases to zero, and clockwise rotation begins without interruption of elongation growth. Stage IVb begins with the initiation of left-handed helical growth. Stage IVb exhibits nearly constant growth zone length (~2.5 mm), elongation growth rates (~35 μm·min^−1^) and rotation growth rates (~12 degrees·min^−1^) for many hours. Stage IVb sporangiophores are typically used for most biophysical experimental studies. Stage IVc is initiated by counter-clockwise rotation and right-handed helical growth. Stage V does not exhibit visible expansive growth. The large cylindrical single-celled sporangiophores are approximately 150 μm in diameter and can grow in length to ten or more centimeters. The sporangiophores can detect many environmental stimuli (e.g., gravitational acceleration, ethylene, mechanical stretch, gases, temperature, wind, light intensity, spatially asymmetric distribution of light, the presence of solid objects and turgor pressure) and respond to these stimuli with symmetric and asymmetric changes in expansive growth rate (e.g., growth responses and tropic responses) [7,17,18].

**Figure 2:**
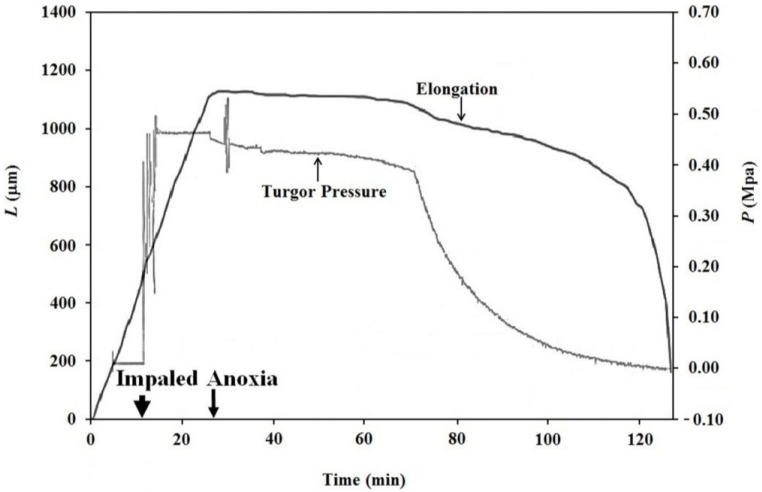
The change in elongation, Δ*L*, and turgor pressure, *P*, of a single Stage IV sporangiophore is plotted against the same time scale. Turgor pressure measurements begin at 10 min on the time scale and are marked by the downward pointing arrow labeled “Impaled”. The turgor pressure curve (gray) is the trace from the strip-chart recorder that measures the output of the pressure transducer in the pressure probe. The oxygen concentration is decreased from 21% to less than 1% at approximately 27 min on the time scale and is marked by the downward pointing arrow labeled “Anoxia”. The immediate small decrease in turgor pressure from a constant value indicates the exact time when the oxygen concentration was decreased to less than 1%. Jessica E.C. Olson and Elena L. Ortega (two graduate students) conducted this experiment while working in Joseph K.E. Ortega’s laboratory.

Growing Stage IV sporangiophores of the fungus *P. blakesleeanus* (Figure 1) were subjected to an anoxic environment in order to terminate the sporangiophore’s metabolism [17,19], thus eliminating the export of wall polymers and materials from the protoplasm to the wall. Experiments were conducted as follows. A Stage IV sporangiophore was selected and placed in a chamber made of transparent acrylic that was constructed specifically for these experiments. The sporangiophore was adapted in the experimental chamber to atmospheric air (~21% oxygen concentration), constant and symmetric distribution of light and other environmental conditions for 20 min. The light and environmental conditions were maintained constant throughout the adaptation period and during the experiment to prevent growth responses. After the adaptation period, the elongation growth was measured for the remainder of the experiment (Figure 2). Ten minutes afterwards, the sporangiophore was impaled with the micro-capillary tip of a pressure probe (downward pointing arrow on the time scale and labeled “Impaled”), and the turgor pressure was measured for the remainder of the experiment. At approximately 27 min on the time scale (shown by the downward point arrow labeled “Anoxia”), the chamber was filled with nitrogen gas, which decreased the oxygen concentration to less than 1%.

The results presented in Figure 2 show that both the elongation rate (slope of the elongation curve, *ΔL*) and turgor pressure (*P*) remain nearly constant between 10 min and 27 min on the time scale. The sharp and immediate decrease in turgor pressure occurs at the exact time when the oxygen concentration decreased from 21% to less than 1%. It can be seen that the turgor pressure continues to decrease for the remainder of the experiment. Interestingly, the turgor pressure decreases slowly for approximately 40 min and then suddenly decreases exponentially. This turgor pressure behavior was typical of more than five experiments conducted.

The elongation decreases to zero within a few minutes after the initiation of anoxia and continues to decrease slowly for another 40–50 min. Afterwards, the elongation decreases faster for the remainder of the experiment until the sporangium falls over onto the stalk. The decrease in elongation represents a decrease in the length of the sporangiophore stalk. The shrinking in length can be explained as “recovered” elastic wall deformation that occurs when the turgor pressure decreases in magnitude. This recovered elastic wall deformation will obscure any irreversible wall deformation (expansive growth) that may occur during anoxia. It is apparent that the turgor pressure must remain constant in order to measure irreversible wall deformation.

**Figure 3:**
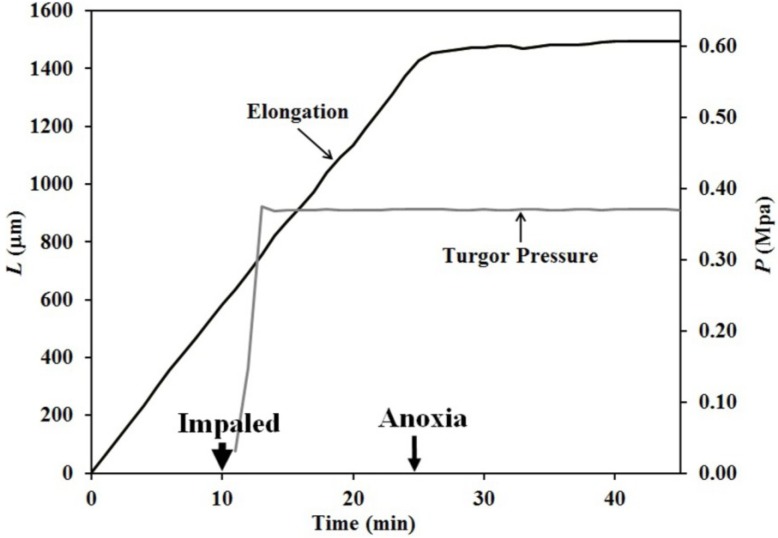
Both the change in elongation and turgor pressure of a Stage IV sporangiophore are plotted as a function of time. The turgor pressure was held constant with the pressure probe during anoxia.

The pressure probe was used to measure and control the turgor pressure in the sporangiophore before and during anoxia. Figure 3 shows the results from one of these experiments. As before, a Stage IV sporangiophore was adapted to constant environmental conditions in the chamber for 20 min before elongation measurements began, and the conditions remained constant for the remainder of the experiment. The turgor pressure measurements began at approximately 12 min on the time scale, and the oxygen concentration was reduced to less than 1% at 25 min on the time scale. The turgor pressure was held constant at the value that was measured before the initiation of anoxia. Careful inspection of the elongation curve shows that elongation continues slowly for 15 min after the initiation of anoxia.

**Figure 4:**
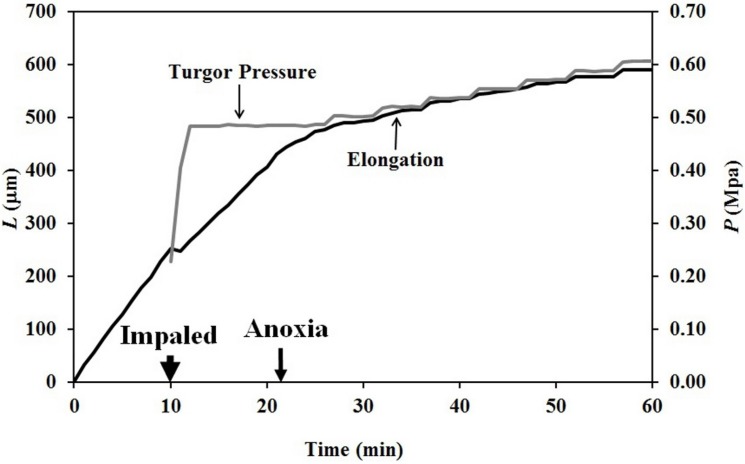
Both the change in elongation and turgor pressure of a Stage IV sporangiophore are plotted as a function of time. Approximately 5 min after anoxia, turgor pressure step-ups (approximately 0.021 MPa in magnitude and 5 min in duration) were produced with the pressure probe.

There was some concern that the oil-cell sap interface within the micro-capillary tip of the pressure probe was unable to move while the turgor pressure was held constant during anoxia. The cell sap of the sporangiophore is very sticky and has a tendency to adhere to the inner wall of the micro-capillary tip and prevents an accurate measurement of the turgor pressure. In order to prevent the oil-cell sap from sticking in one location, small step-ups in turgor pressure were produced by injecting inert silicon oil inside the micro-capillary tip into the vacuole of the sporangiophore (Figure 4). As can be seen, turgor pressure measurements began at approximately ten minutes on the time scale, and an anoxic environment was produced at 22 min. Careful inspection of the elongation curve reveals that elongation growth occurred 2–5 min after each turgor pressure step-up (when *P* = constant) until approximately 45 min. The elongation that occurs one minute after the step-up in turgor pressure is predominately an elastic response and is not used to determine elongation growth and irreversible wall deformation. For this experiment, it is concluded that elongation and irreversible wall deformation continue for 23 min after anoxia.

**Figure 5:**
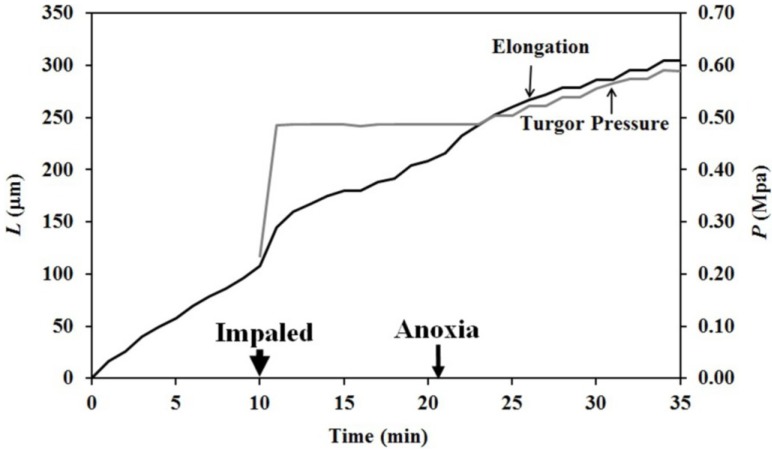
Change in elongation and turgor pressure for a Stage I sporangiophore are plotted as a function of time. Two minutes after anoxia, turgor pressure step-ups (approximately 0.021 MPa in magnitude and 2 min in duration) were produced with the pressure probe.

Similar step-up experiments were conducted on Stage I sporangiophores (Figure 5). The Stage I sporangiophore was impaled to measure the turgor pressure at 10 min on the time scale, and anoxia was produced at 21 min. Inspection of the elongation curve shows that elongation growth and irreversible wall extensibility continued until approximately 27 min, or 6 min after anoxia. It was found that the duration of elongation growth and irreversible wall deformation after anoxia was shorter for Stage I sporangiophores compared to Stage IV sporangiophores.

Some of the results of these experiments are summarized in Table 1, which compares the “duration of elongation during anoxia” obtained from Stage IV sporangiophores when the turgor pressure is held constant and when the turgor pressure is stepped up. Results of Student’s *t*-test indicate that there is not a significant difference between the values obtained when *P* was held constant and when *P* was stepped-up. Because the values are statistically the same, they are combined. The results indicate that, on average, the Stage IV sporangiophore continues to exhibit elongation growth and irreversible wall deformation for approximately 17 min during anoxia. Because the Student’s *t*-test indicates that there is no significant difference between the durations obtained from experiments when *P* was constant and when *P* was stepped up, only the step-up in *P* experiments was conducted for Stage I sporangiophores. The results presented in Table 1 indicate that the duration of elongation growth for Stage I sporangiophores is smaller than the corresponding values obtained from Stage IV sporangiophores. Student’s *t*-tests confirm this observation: the duration of elongation during anoxia of Stage I sporangiophores is significantly smaller (*p* < 0.05) than that obtained from Stage IV sporangiophores when *P* was constant, when *P* was stepped up and when the values were combined.

**Table 1:** Duration of elongation growth during anoxia for Stage IV and Stage I sporangiophores.

Stage	Turgor Pressure during Anoxia	Elongation Duration during Anoxia (min)
IV	*P* = constant	14.8 ± 2.1 SE (*n* = 5)
IV	Step-up in *P*	18.7 ± 3.7 SE (*n* = 6)
IV	Combined	16.9 ± 2.1 SE (*n* = 11)
I	Step-up in *P*	10.0 ± 1.2 SE (*n* = 9)

### 2.2. Frozen-Thawed Sporangiophores

**Figure 6:**
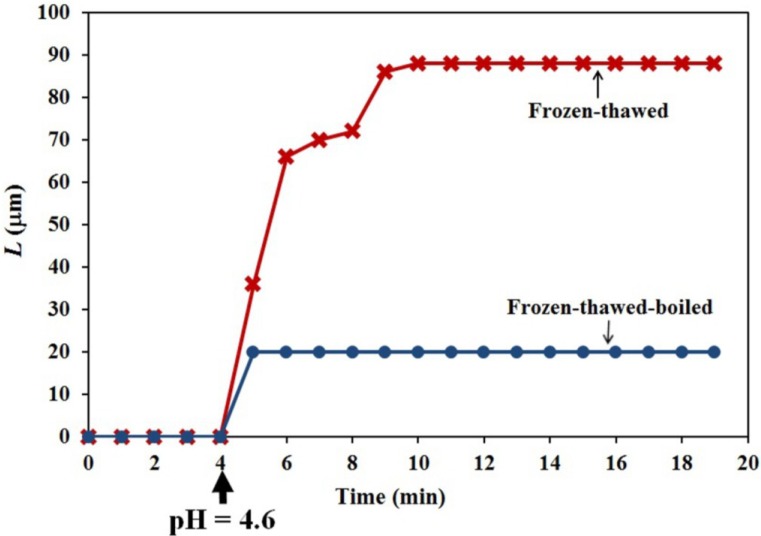
Extension behavior of two 5 mm-long sections from two different Stage IV sporangiophores that were frozen and then thawed is shown. Each wall section includes the growth zone of a Stage IV sporangiophore. The red curve with X data points and labeled “Frozen-thawed” shows the extension behavior of a frozen-thawed wall before and after the pH of the bathing solution was reduced from 7.0 to 4.6. The blue curve with • data points and labeled “Frozen-thawed-boiled” shows the extension behavior of a frozen-thawed wall that was boiled in water for 15 s before being subjected to the same experimental protocol.

Constant-tension extension experiments were conducted on frozen and then thawed walls of the Stage IV sporangiophore to obtain insight into the wall chemistry and chemorheological process. A five millimeter-long section of the wall from a Stage IV sporangiophore, which includes the growth zone, was adapted in an experimental chamber to a bathing solution of pure water (70 mL, pH = 7, at 20 °C) for 20 min. Then, a tensile load of 1.24 g was applied to the wall. The tensile load produces longitudinal stress and longitudinal extension of the wall. After the extension, the length remained constant for four minutes without creep; see Figure 6 (0–4 min). At four minutes on the time scale, the pH of the solution was lowered to 4.6 by adding a predetermined amount of pH Red 4.0 buffer to the bathing water. Immediately, the wall extends (red curve with X data points and labeled “Frozen-thawed”) and continues to extend for six minutes (4–10 min), exhibiting five minutes of creep (5–10 min). Also shown in Figure 6 are the results of another experiment where the same experimental protocol was conducted on another frozen-thawed wall, except that the frozen-thawed wall was immersed in boiling water for 15 s before being subjected to the experimental protocol (blue curve with • data points and labeled “Frozen-thawed-boiled”). It can be seen that the wall extends after the pH of the bathing solution was reduced to 4.6; however, the initial extension is small, and the wall does not continue to extend afterwards, *i.e.*, it does not creep.

**Figure 7:**
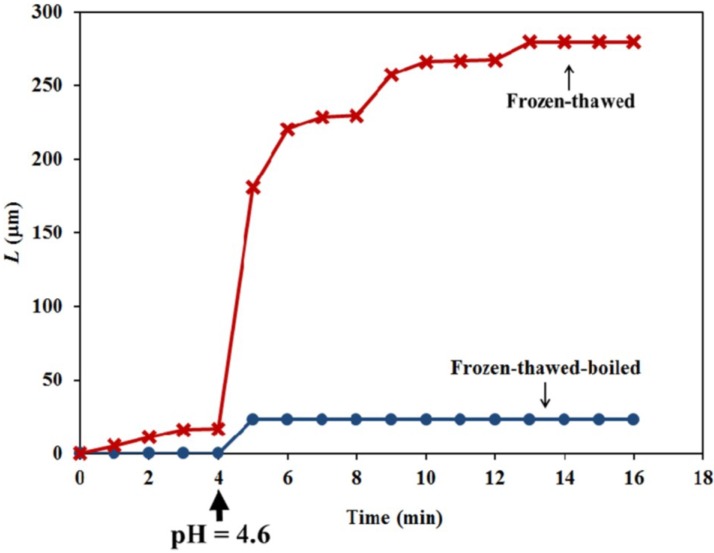
The average change in extension *versus* time of 15 different frozen-thawed walls is shown (red curve with X data points and labeled “Frozen-thawed”). The average change in extension *versus* time of eight frozen-thawed-boiled walls (blue curve with • data points and labeled “Frozen-thawed-boiled”) is shown.

These experiments were repeated many times. Figure 7 is a plot of the average change in extension at each minute on the time scale of 15 different frozen-thawed walls (red curve with X data points and labeled “Frozen-thawed”) and eight frozen-thawed-boiled walls (blue curve with • data points and labeled “Frozen-thawed-boiled”). The results plotted in Figure 7 indicate that the frozen-thawed walls creep for eight minutes (5–13 min) after the pH of the bathing solution is reduced from 7.0 to 4.6.

Student’s *t*-tests were conducted to determine whether the extension rates obtained from frozen-thawed walls after the pH is lowered to 4.6 are significantly different from those of frozen-thawed-boiled walls. The results are shown in Table 2. The results indicate that the extension rates for the frozen-thawed walls are significantly higher compared to the frozen-thawed-boiled walls (*p* < 0.05) for 2–5 min after the pH was reduced. The interpretation is that creep occurs for four minutes (2–5 min) after the pH was reduced to 4.6. Fewer results indicate that in some experiments, there is creep until the ninth minute after the pH is lowered to 4.6.

**Table 2:** Extension rate for each minute and the result of the Student’s *t*-tests for frozen-thawed and frozen-thawed-boiled cell walls.

Minutes after pH = 4.6	Frozen-Thawed Extension Rate (μm/min)	Frozen-Thawed-Boiled Extension Rate (μm/min)	Student’s *t*-Test
1	191 ± 109.1 SE (*n* = 13)	23.3 ± 2.8 SE (*n* = 8)	*p* = 0.77
2	74.6 ± 32.7 SE (*n* = 9)	0	*p* = 0.024
3	17.6 ± 5.4 SE (*n* = 7)	0	*p* = 0.005
4	6.0 ± 2.8 SE (*n* = 3)	0	*p* = 0.018
5	59.6 ± 27.1 SE (*n* = 7)	0	*p* = 0.024
6	43.0 ± 45.9 SE (*n* = 3)	0	*p* = 0.154
7	10.0 (*n* = 1)	0	------
8	5.0 (*n* = 2)	0	------
9	181.0 (*n* = 1)	0	------

## 3. Discussion

### 3.1. Anoxia Experiments

Anoxia was used to chemically isolate the wall from the protoplasm of the sporangiophores of *P. blakesleeanus* and to reveal the wall chemistry and chemorheology that produces irreversible wall extension and elongation growth. The sporangiophores of *P. blakesleeanus* strictly require oxygen for growth and development [17,19]. The relationship between elongation growth and the metabolism of the sporangiophores was studied by using anoxia to inhibit oxidative phosphorylation [17]. Bergman *et al.* [17] report, “Many fungi can grow anaerobically for long periods of time, but *Phycomyces* sporangiophores do not have this capacity. When oxygen is removed from sporangiophores by placing them in nitrogen, streaming and growth rapidly stop.” Studies conducted in our laboratory by Morgan A. Scott (Master’s report, see Acknowledgments) show that streaming inside the sporangiophore stops within a minute; *t* = 39.5 ± 2.4 (SE) s, *n* = 10. The finding that the turgor pressure continually decreases after the initiation of anoxia is consistent with the results of earlier studies that anoxia terminates the protoplast’s metabolism.

The finding that elongation growth continues during anoxia for ten minutes or longer when the turgor pressure is held constant with the use of a pressure probe demonstrates that a wall chemistry continues the chemorheological process to produce irreversible wall deformation during anoxia. The wall deformation is irreversible, because the elongation growth occurs when the turgor pressure is constant (Figure 3, Figure 4 and Figure 5). Inspection of the previously-established augmented growth equation, Equation (2), can show this. The augmented growth equation can be modified to describe the elongation growth of cells with a growth zone, such as the sporangiophores of *P. blakesleeanus*; Equation (3) [20].

(3) $$\frac{dL}{dt}=m_{g}\;\left(P-P_{C}\right)+\frac{L_{g}}{E_{g}}\frac{dP}{dt}+\;\frac{L_{s}}{E_{s}}\frac{dP}{dt}$$

*Elongation rate = irreversible rate in growth zone + elastic rate in growth zone + elastic rate in stalk*

where *L* is the length of the sporangiophore, d*L*/d*t* is the elongation rate, *m*_g_ is the longitudinal irreversible wall extensibility of the growth zone, *P* is the turgor pressure, *P*_C_ is the critical turgor pressure and *L*_g_ and *L*_s_ are the lengths of the growth zone and stalk, respectively. *E*_g_ and *E*_s_ are the longitudinal components of the volumetric elastic modulus within the growth zone and non-growing stalk, respectively. The term, *m*_g_ (*P − P*_C_), represents the longitudinal irreversible deformation rate of the wall in the growth zone; the term, (*L*_g_/*E*_g_) d*P*/d*t*, represents the longitudinal elastic deformation rate of the wall in the growth zone; and the term, (*L*_s_/*E*_s_) d*P*/d*t*, represents the longitudinal elastic deformation rate of the wall in the non-growing stalk.

It is noted in Equation (3) that when the turgor pressure is constant (*P* = constant, then d*P*/d*t* = 0), the elongation rate is only a function of the irreversible longitudinal deformation rate of the wall in the growth zone; Equation (4). Equation 4 is essentially the same equation previously derived by Lockhart [21]. Thus, the elongation rate that occurs when *P* = constant (for 15 min after the initiation of anoxia in Figure 3, between 2–5 min after the first five turgor pressure step-ups in Figure 4 and after the first two turgor pressure step-ups in Figure 5) represents the irreversible wall deformation rate.

(4) $$\frac{dL}{dt}=m_{g}\;\left(P-P_{C}\right)$$

*Elongation rate = irreversible longitudinal deformation rate in growth zone*

The irreversible wall deformation and elongation growth that occur during anoxia are direct evidence of a wall-loosening chemistry or chemorheological process. In the sporangiophores, the wall chemistry and chemorheological process continue for a limited duration during anoxia, typically 10–20 min. Two explanations for the limited duration of elongation growth and associated wall-loosening chemistry during anoxia are considered. The first explanation is that irreversible wall deformation continues during the wall hardening process that begins at the initiation of anoxia. The wall hardening process converts an irreversible extensible wall to a reversible extensible wall by making bonds between microfibrils and other wall polymers. It is predicted that the irreversible wall deformation stops when the wall hardening process is complete. Second, wall building polymers and other relevant wall materials extruded into the periplasm and inner wall surface before the initiation of anoxia, are used to continue the wall-loosening chemistry and maintain the irreversible wall extensibility. After these polymers and materials are depleted, then the wall hardening process begins and continues until completion.

The first explanation predicts that the duration of elongation growth during anoxia for Stage I and Stage IV sporangiophores are approximately the same, because it is expected that the wall hardening process for both stages is similar and the rate of hardening is approximately the same. The second explanation predicts that the duration of elongation growth for Stage IV sporangiophores is larger than those of Stage I sporangiophores, because the elongation growth rates of Stage IV sporangiophores (34.1 ± 3.1 (SE) μm·min^−1^, *n* = 20) are significantly larger than those of Stage I sporangiophores (7.1 ± 0.7 (SE) μm·min^−1^, *n* = 17) [7]. The larger elongation growth rates of Stage IV sporangiophores require a faster delivery rate of wall-building polymers and relevant wall-building materials to the periplasm and inner wall surface compared to Stage I sporangiophores. It is reasonable to expect that the amount of wall-building polymers and relevant wall-building materials within the periplasm and inner wall surface at the time anoxia is initiated (and the wall is chemically isolated) is larger for Stage IV sporangiophores compared to Stage I sporangiophores. If the amount of wall building substrates for the wall chemistry is larger for Stage IV, it is predicted that the duration of the wall chemistry, wall loosening, irreversible deformation and elongation growth will be longer for Stage IV compared to Stage I. The second prediction is consistent with the results presented in Table 1.

### 3.2. Frozen-Thawed Sporangiophores

Extension experiments were conducted on frozen-thawed sporangiophore walls to obtain insight into the wall loosening chemistry. The constitutive equation for a Maxwell–Bingham viscoelastic model, Equation (1), can be modified to assist in the interpretation of the results. Equation (5) is derived from Equation (1) and describes the longitudinal deformation (extension) rate of the frozen-thawed wall section after a tensile load, or longitudinal stress (*σ*), is applied.

(5) $$\frac{dL}{dt}=m_{f}\;\left(\sigma -\sigma_{C}\right)+\frac{L_{o}}{E_{f}}\frac{d\sigma }{dt}$$

*Longitudinal deformation rate = irreversible deformation rate + elastic deformation rate*

where *L* is the length of the frozen-thawed wall section, d*L*/d*t* is the longitudinal deformation (extension) rate, *m*_f_ is the longitudinal irreversible wall extensibility of the frozen-thawed growth zone wall section, *σ* is the longitudinal stress, *σ*_C_ is the critical longitudinal stress, *L*_o_ is the initial length and *E*_f_ is the longitudinal elastic modulus. The term, *m*_f_ (*σ – σ*_C_), represents the longitudinal irreversible deformation rate of the wall in the growth zone, and the term, (*L*_o_/*E*_f_) d*σ*/d*t*, represents the longitudinal elastic deformation rate of the wall in the growth zone.

Equation (5) is similar to an equation previously used by Takahashi *et al.* [22] to analyze the relationship between the extension behavior of frozen-thawed tissue sections of cucumber hypocotyls and expansin. Here, in Equation (5), we explicitly recognize that *m*_f_ (longitudinal irreversible wall extensibility that results from unidirectional stress) is not equal to *ϕ* or *m*_g_ (irreversible wall extensibility or the longitudinal component of the irreversible wall extensibility, respectively, that results from multidirectional stresses produced by turgor pressure *in vivo*), and *E*_f_ (the longitudinal elastic modulus that results from unidirectional stress) is not equal to *ε* (the volumetric elastic modulus that results from multidirectional stresses produced by turgor pressure *in vivo*).

Constant tension-extension experiments were conducted using a tensile force of 1.24 gf, or 0.012 N (*F* = mass × gravitational acceleration = (1.24 × 10^−3^ kg) (9.81 m·s^−2^) = 0.012 N) that was applied to the frozen-thawed walls. The applied longitudinal wall stress can be estimated using an average sporangiophore diameter of *D* = 150 µm and a wall thickness of *τ* = 0.6 µm; *σ* = *F*/π*Dτ* ≅ 42 MPa. Immediately after applying the longitudinal wall stress (tensile force of 0.012 N), initial longitudinal wall deformations (extensions) ranged from 1–2 mm. The initial wall extension in response to the application of the wall stress can be determined using Equation (5), where d*σ*/d*t* is finite and relatively large (typically, the wall stress of 42 MPa is applied within a few seconds). It is apparent that the initial longitudinal extensions are predominately elastic; d*L*/d*t* = (*L*_o_/*E*_f_) d*σ*/d*t*. After the initial longitudinal extension, the longitudinal stress and length of the frozen-thawed wall section remained constant. Because *σ* is constant in Equation (5) (*i.e.*, d*σ/*d*t* = 0), subsequent extensions that occur after decreasing the pH of the bathing solution to 4.6 represent irreversible deformations and creep, Equation (6).

(6) $$\frac{dL}{dt}=m_{f}\;\left(\sigma -\sigma_{C}\right)$$

*Longitudinal deformation rate* = *Irreversible deformation rate*

The applied longitudinal wall stress, *σ* ≅ 42 MPa, is larger than the estimated longitudinal stress produced by the turgor pressure *in vivo*. Previously, the pressure probe was used to determine the average turgor pressure (*P* = 0.32 ± 0.01 (SE) MPa, *n* = 20) and average critical turgor pressure (*P*_C_ = 0.26 ± 0.01 (SE) MPa, *n* = 20) ([7] and the references within). The longitudinal stress produced by turgor pressure can be estimated using the equation, *σ* = *PR*/2*τ*, where *P* is the turgor pressure, *R* is the radius (75 µm) and *τ* is the wall thickness (0.6 µm). The longitudinal wall stress produced by the average turgor pressure (*P* = 0.32 MPa) is estimated to be, *σ* = 20 MPa, which exceeds the estimated critical wall stress produced by the average critical turgor pressure (*P*_C_ = 0.26 MPa), *σ*_C_ ≅ 16.3 MPa.

The objective of the constant-tension extension experiments conducted on frozen-thawed sporangiophore walls is to learn if lowering the pH of the bathing solution will elicit irreversible extension and creep, similar to what was found in higher plant cells. The results shown in Figure 6 and Figure 7 (red curve with X data points and labeled “Frozen-thawed”) demonstrate that lowering the pH of the bathing solution from 7.0 to 4.6 elicits a chemorheological process, irreversible wall extension (see Equation (6)) and wall creep. In contrast, if the wall sections were immersed in boiling water for 15 s before the constant tension-extension experiments, they did exhibit a nearly instantaneous irreversible deformation, but did not exhibit creep, after the pH of the bathing solution was reduced to 4.6.

Constant-tension extension experiments were previously conducted on frozen-thawed sections of growing cucumber (*Cucumis sativus* L.) hypocotyls and oat (*Avena sativa* L.) coleoptiles immersed in a bathing solution [10,11]. When the pH of the bathing solution was reduced to 4.5, the frozen-thawed walls begin to extend, and the extension continued for hours afterwards, thus exhibiting creep. The experimental results are consistent with the “acid growth” hypothesis [9], which states that plant cells excrete protons onto the inner surface of the cell wall to decrease the pH and activate an “unknown” wall loosening process. When the frozen-thawed cucumber and oat walls were boiled in water for 15 s before the extension experiment, the walls did not exhibit creep when the pH of the bathing solution was lowered to 4.5. These and other experimental results suggested that protein activity may mediate wall loosening at low pH. Subsequently, wall-loosening proteins were isolated that are not enzymes, expansins [1]. Expansins are hypothesized to catalyze wall stress relaxation and irreversible wall deformation directly by disrupting hydrogen bonds between the cellulose microfibrils and hemicellulose [23]. Expansins have been found in a variety of plants and in bacteria and fungi [1,12]. Crude extracts and purified expansins have been shown to mediate “acid-induced” extension of boiled isolated primary walls of a variety of plants [1]. Wall loosening by expansins is consistent with the acid growth hypothesis. Expansins are thought to be strong candidates for primary wall-loosening agents for cells in the organs of higher plants (e.g., stems, roots and leaves). However, endogenous wall enzymes, such as xyloglucan endotransglycosylase/hydrolase and endo-β-1,4-glucanases, are also hypothesized to play an important role in expansive growth of higher plants [13]. Importantly, a considerable amount of experimental evidence indicates that pectin plays a major role in the expansive growth of pollen tubes ([14] and the references within) and algae ([15] and the references within). Furthermore, pectin may play a role in the expansive growth of higher plant cells [3,8]. Interestingly, a small amount of pectin is found in the sporangiophore’s cell wall [24], and one wonders if pectin plays a role in the expansive growth of sporangiophores and fungal cells in general.

The general structure of the sporangiophore’s wall can be described as chitin and β-glucans microfibrils embedded in an amorphous matrix composed predominately of chitosan, glycoproteins and lipids [16,18], with a small amount of pectin [24]. The wall polymers are linked together by covalent bonds, hydrogen bonds, hydrophobic interaction and ionic associations [16]. The microfibrils are extruded onto the inner wall surface by chitin synthases embedded in the plasma membrane [16]. Chitin synthases and other enzymes and wall polymers are transported to the plasma membrane in vesicles (chitosomes) and delivered to the periplasm via exocytosis [16]. Importantly, fibrillogenesis and microfibril networks have been shown to occur *in vitro* by incubating purified chitin synthases with substrate (UDP-GLcNAc) and activators [25,26]. Furthermore, more recently, the genome of *P. blakesleeanus* was sequenced, and genes involved in sensory growth responses and cell wall components are being characterized (http://genome.jgi-psf.org/Phybl2/Phybl2.home.html).

Generally, the microfibrils can be cross-linked directly (by hydrogen bonds, hydrophobic interaction and ionic associations) and by matrix polymers (chitosan, glycoproteins, and pectin). We hypothesize that wall chemistry and molecular agents that effectively break and make load-bearing cross-links between microfibrils produce chemorheology that regulates wall mechanical properties and wall deformation behavior. Breaking the cross-links directly, or by disconnecting the microfibrils from the matrix network and/or each other, will allow the microfibrils to separate and/or slide passed each other when the wall is stressed by turgor pressure *in vivo* and by an applied stress in extension experiments. In turn, this chemorheological process can produce a controlled polymer creep, often referred to as “wall loosening”. Load-bearing cross-links that are not broken or that were broken and reconnected (after slippage and/or separation of microfibrils) and subsequently become load-bearing produce the elastic behavior of the wall. The wall loosening will initiate wall stress relaxation and turgor pressure relaxation that, in turn, produces water uptake, irreversible wall deformation and an increase in cell volume.

The extension curves obtained from frozen-thawed and frozen-thawed-boiled sporangiophore walls after the pH is lowered to 4.6 are qualitatively similar to those obtained for cucumber (*Cucumis sativus* L.) hypocotyls and oat (*Avena sativa* L.) coleoptiles [10,11]. The experimental results from the frozen-thawed sporangiophore walls are consistent with the “acid growth” hypothesis [9]. An important difference is that the duration of the creep for the sporangiophores’ walls is limited to less than ten minutes, compared to hours for cucumber hypocotyls and oat coleoptiles. The finding that boiling the sporangiophore walls for 15 s eliminates creep may suggest that protein activity mediates the creep response, but other explanations must also be considered until more experiments are conducted.

The finding that the duration of the creep for the sporangiophores’ walls is limited to less than ten minutes, compared to hours for cucumber hypocotyls and oat coleoptiles, is interesting and probably significant. This finding, together with the fact that the molecular polymers, structure and endogenous enzymes in the sporangiophore’s wall are different from those in plant and algal cell walls, may indicate that the molecular wall loosening mechanism is different for fungal cells. It may be significant that the duration of elongation growth during anoxia and the duration of creep in frozen-thawed walls sections are both on the order of ten minutes. Interestingly, the duration of creep produced by decreasing the pH is approximately the same as the duration of the light growth response and avoidance growth response (transient increases in elongation growth rate) exhibited by the sporangiophores [7,17,18]. A mechanism is suggested by these findings in which proton effluxes mediate sensory growth responses of the sporangiophores. One may speculate that the sporangiophore’s protoplast excretes protons at an increased rate onto the inner surface of the growth zone wall to elicit a transient increase in irreversible wall extensibility [27,28] that produces a transient increase in elongation growth rate, without increasing the turgor pressure [29]; see Equation (4). The phototropic response (growing towards a light source) and avoidance response (growing away from solid objects) could be produced by spatially increasing the proton efflux on the distal side (phototropic response) and proximal side (avoidance response) of the sporangiophore’s growth zone. Future research will include adding wall building substrates, activators and endogenous wall enzymes to the bathing solution during constant-tension extension experiments to learn if they will increase the duration of creep in the frozen-thawed sporangiophore wall.

## 4. Experimental Section

### 4.1. Biological Material

The wild-type strain, NRRL1555(−), of *P. blakesleeanus* was obtained from the Biology Division, California Institute of Technology, Pasadena, USA. Potato dextrose agar was prepared and sterilized in 12 × 35 mm glass vials. Vegetative spores were heat-shocked in a water bath held at approximately 45 °C for 20 min. After heat-shocking, the spores were triturated and inoculated on the potato dextrose agar in the glass vials. The sporangiophore cultures in the vials were kept at 21 °C ± 0.5 °C in a ventilated, wood chamber and illuminated from above by four fluorescent lamps. Three days after inoculation, sporangiophores begin to appear. Long sporangiophores are removed from the mycelium (plucked) daily to obtain big and robust sporangiophores for experimentation on the next day. Typically sporangiophores from 4 to 6 day-old cultures are used for experimentation.

### 4.2. Anoxia Experiments

#### 4.2.1. Elongation Growth Measurements

The elongation growth is determined by measuring the change in length of the sporangiophore, Δ*L*, at one-minute time intervals. The change in length is measured using a long focal length horizontal microscope (Gaertner; 7011K eyepiece and 32 m/m EFL objective) mounted to a 3D micromanipulator (Line Tool Co., Allentown, PA, USA; Model H-2, with digital micrometers). An electronic timer is used to measure the time intervals.

#### 4.2.2. Turgor Pressure Measurements

The turgor pressure of the sporangiophore is measured with a manual version of the pressure probe [20]. A gage pressure transducer is used in the pressure probe, which measured the difference between the absolute pressure and the local atmospheric pressure; the gage pressure transducer was purchased from Kulite Semiconductor Products, Ridgefteld, NJ, USA (Model XT-190-300G), and calibrated inside the pressure probe using a Heise Bourdon Tube Pressure Gauge (Dresser Industries, Newton, CT, USA; Model CMM, 0-200 PSIG Range). The transducer’s output is recorded on a Houston Omniscribe Stripchart Recorder (Ametek, Berwyn, PA, USA; Model D5217-2). The pressure probe was mounted on a 3D micromanipulator so that the micro-capillary tip (typically 5–10 μm outer diameter) can be guided to impale the sporangiophore under visual observation using a horizontally-mounted EZM-2TR Trinocular Zoom Stereomicroscope (Meiji Labax Co., Tokyo, Japan). The micro-capillary of the pressure probe was filled with inert silicone oil (Dow Corning Corp., Midland, MI, USA; fluid 200, 1–2 centistoke viscosity). After the cell was impaled, the cell sap-oil interface was maintained at a fixed location within the micro-capillary tip to measure the turgor pressure of the sporangiophore. Turgor pressure step-ups were produced by advancing a manually-controlled control rod within the pressure probe to inject inert silicone oil into the vacuole.

#### 4.2.3. Experimental Protocol

**Figure 8:**
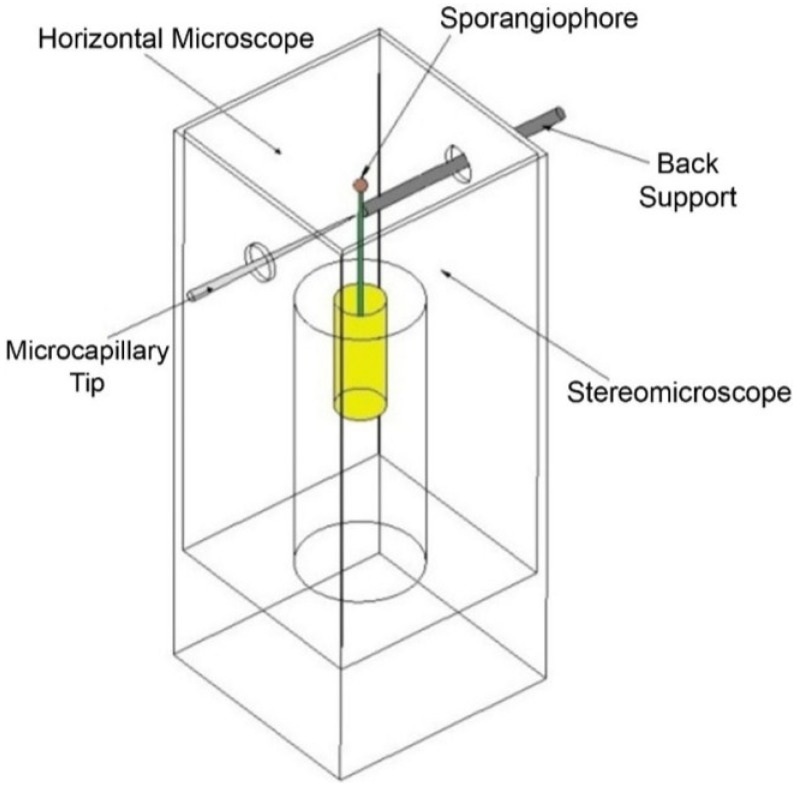
An illustration of the experimental chamber used for anoxia experiments. A vial containing a sporangiophore is inserted into a holder inside the chamber. Afterwards, a back support is advanced manually to the sporangiophore stalk. The back support keeps the stalk stationary while the micro-capillary tip of the pressure probe is advanced to impale the stalk below the growth zone*.* Two people are required to conduct a single experiment. One person uses a stereomicroscope to impale the sporangiophore stalk, monitor the cell sap-oil interface and generally operate the pressure probe. On the opposite side, another person uses a horizontal microscope, attached to a 3D micromanipulator with digital micrometers, to measure the change in elongation as a function of time.

A Stage IV sporangiophore was selected and placed in an experimental chamber that was constructed for these experiments (Figure 8). The sporangiophore was adapted in the experimental chamber to atmospheric air, constant and symmetric distribution of light and other environmental conditions for 20 min. The light and environmental conditions were maintained constant throughout the adaptation period and during the experiment to prevent growth responses. After the adaptation, measurement of the elongation growth was initiated and continued for the remainder of the experiment using a long focal length horizontal microscope. After a period of time (usually 10 min), the sporangiophore stalk was impaled with the pressure probe’s micro-capillary tip with the assistance of a horizontally-mounted stereomicroscope, and the turgor pressure was continuously measured and monitored with the pressure probe for the remainder of the experiment. Then, both the elongation rate and turgor pressure were monitored for a period of time to ensure that the sporangiophore was growing at a normal rate. Afterwards, nitrogen gas was injected from the bottom of the chamber to fill the apparatus chamber containing the sporangiophore and to create an anoxic environment. The oxygen concentration was measured and monitored with a meter (Model 50SD-2, 0–20 mL/min, McMillan Company, Georgetown, TX, USA) inside the chamber and located next to the sporangiophore’s growth zone. Typically, the oxygen concentration decreased from approximately 21% to less than 1% within a minute after the nitrogen gas was injected into the chamber. Nitrogen gas was continuously injected into the chamber at a low rate to maintain an oxygen concentration level of less than 1%.

### 4.3. Frozen Thawed Experiments

Sporangiophores, which are frozen for one or two days and then thawed, were used for these experiments. A 5-mm section from the sporangiophore stalk that contains the growth zone was cut with a razor blade and used for experimentation.

#### 4.3.1. Experimental Apparatus

**Figure 9:**
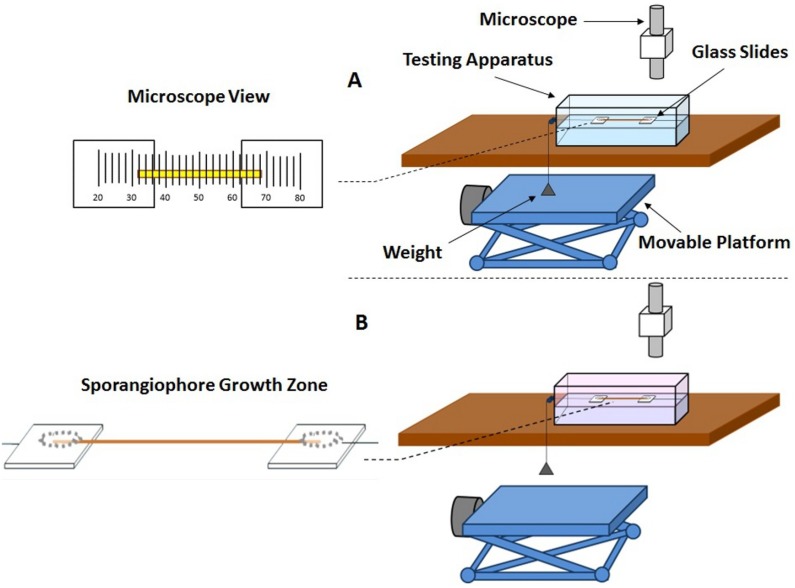
Schematic illustration of the experimental set-up used for the constant-tension extension experiments. (**A**) Testing arrangement for the frozen-thawed wall section during the adaptation phase. The testing apparatus is filled with water, and the platform supporting the weight is not lowered (right side). A microscope view of the aligning process (left side) of the frozen-thawed wall section to the reference point in the microscope, so that changes in length can be measured during the experiment by realigning to the reference point every minute and measuring the change in length on the digital micrometer; (**B**) Testing arrangement for the frozen-thawed wall section during the extension testing phase. The platform is lowered, and the weight hangs freely during the test (right side). Buffer solution (pink) is added to the testing apparatus to lower the pH to 4.6. A microscope view of the wall section in tension is depicted (left side).

A small acrylic rectangular box was constructed and used as the testing apparatus for the extension experiments (Figure 9). In the testing apparatus, the frozen-thawed wall section is attached to two glass slides, one at each end (glued with Gorilla Super Glue). One glass slide is attached to the testing apparatus and fixed. The other glass slide is attached to a small weight (1.24 g) with a string and is free to slide within the box. The weight is supported on a platform to prevent tension on the wall section before the extension protocol is initiated. A microscope assembly attached to a micrometer is used to measure the change in length of the wall section (Figure 9A). A reference point (end of the wall section) is used throughout the experiment to measure the change in length of the wall section as a function of time. Initially, the frozen-thawed wall section is aligned to the reference point in the microscope, so that changes in length can be measured during the experiment by realigning to the reference point every minute and measuring the change on the digital micrometer.

#### 4.3.2. Experimental Protocol

A Stage IV sporangiophore is selected and frozen for at least 24 h. A 5-mm wall section that includes the growth zone of the frozen sporangiophore is removed with a razor blade. Then, each end of the wall section is glued to a glass slide inside the testing apparatus, one of which is fixed (stationary) and the other (attached to a weight by a string) is free to slide. The frozen wall section thaws while the glue dries. After the glue is dry and the wall section is securely clamped to the two glass slides, distilled water is added to the apparatus until the top part of the apparatus is half full and left to adapt for 10 min (Figure 9A). Following this adaptation period, the change in length is measured every minute for the remainder of the experiment, and the platform supporting the weight is slowly lowered to apply tension to the wall section until the weight hangs freely. Five minutes after the wall section has been in constant tension, a predetermined amount of buffer solution was added to the water to lower the pH to approximately 4.6. A pH pen (Large Display ATC pH Pen, Model 850051, VWR International) is used to measure the pH of the bathing solution when the buffer solution is first added and at the end of the experiment. The change in length is measured continuously until no extension is observed (Figure 9B). The experimental protocol for the frozen-thawed-boiled experiments is the same, except that after removing the sporangiophore from the freezer, it is transferred into a water bath and boiled for 15 s using a microwave before the extension protocol is conducted.

## 5. Conclusions

Experiments that used anoxia to terminate the metabolism of the protoplasm of Stage I and Stage IV sporangiophores of *P. blakesleeanus* and chemically isolate their walls reveal a wall chemistry and chemorheology that continues for ten minutes or longer to produce elongation and irreversible wall extension (Figure 3, Figure 4, Figure 5 and Table 1). Constant-tension extension experiments were conducted on frozen-thawed wall sections of Stage IV sporangiophores to investigate the wall chemistry and chemorheology revealed in the anoxia experiments. The objective was to learn if lowering the pH of the bathing solution from 7.0 to 4.6 will elicit irreversible extension and creep. The results (Figure 6, Figure 7 and Table 2) demonstrate that lowering the pH produces irreversible wall extension and creep in the frozen-thawed wall sections (which include the growth zone) of the Stage IV sporangiophore. This finding is consistent with the “acid growth” hypothesis that was proposed for plant cell walls [9]. Interestingly, frozen-thawed wall sections that were subject to boiling water for 15 s do not exhibit creep after the pH is lowered to 4.6. This finding suggests that protein activity may be involved in the observed creep response, but other experimental results are needed to support this suggestion. Except for the short duration of creep, the constant tension-extension behavior of the frozen-thawed and frozen-thawed-boiled wall sections of the Stage IV sporangiophore is qualitatively similar to that obtained from cucumber (*Cucumis sativus* L.) hypocotyls and oat (*Avena sativa* L.) coleoptiles [10,11]. It is thought that the limited and short duration of creep exhibited by the frozen-thawed sporangiophore wall to acidic pH may be significant. This finding, together with the fact that the molecular polymers, structure and endogenous enzymes in the sporangiophore walls are different from those in plant and algal cell walls, may suggest that the molecular wall loosening mechanism is different from those of higher plant cells and algal cells.
